# SIRT3 inhibits autophagy-dependent ferroptosis of HUVECs in the progression of pregnancy-induced hypertension by activating the PI3K/Akt/mTOR axis

**DOI:** 10.3389/fnmol.2025.1620184

**Published:** 2025-10-03

**Authors:** Yanchuan Li, Yanfeng Liu, Ziyun Shi, Meiqing He

**Affiliations:** ^1^Department of Obstetrics, Shaanxi Provincial People’s Hospital, Xi’an, Shaanxi, China; ^2^Department of General Surgery, The Second Affiliated Hospital of Xi'an Medical University, Xi’an, Shaanxi, China; ^3^Department of Ultrasound, Shaanxi Provincial People’s Hospital, Xi’an, Shaanxi, China

**Keywords:** pregnancy-induced hypertension, autophagy, ferroptosis, SIRT3, PI3K/Akt/mTOR axis

## Abstract

**Introduction:**

Pregnancy-induced hypertension (PIH) is a major contributor to increased maternal and neonatal morbidity and mortality worldwide. Accumulating evidence suggests that vascular endothelial cell injury is a key pathological feature in the development of PIH; however, the underlying molecular mechanisms remain incompletely understood. This study aimed to investigate the role of sirtuin 3 (SIRT3) in regulating autophagy and ferroptosis in endothelial cells under hypoxic conditions, and its potential impact on PIH pathogenesis.

**Methods:**

Human umbilical vein endothelial cells (HUVECs) were subjected to hypoxia/reoxygenation (H/R) to mimic ischemic-reperfusion injury associated with PIH. Autophagy levels were assessed by measuring the ratio of microtubule-associated protein light chain 3 (LC3)-II/LC3-I and sequestosome 1/p62 expression. Ferroptosis markers, including intracellular iron, reactive oxygen species (ROS), malondialdehyde (MDA), glutathione (GSH), and glutathione peroxidase 4 (GPX4), were evaluated. SIRT3 was overexpressed using pcDNA-SIRT3, and autophagy was modulated with rapamycin or SIRT3 overexpression. The involvement of the PI3K/Akt/mTOR pathway was examined by Western blotting. In vivo validation was performed using a rat model of PIH with SIRT3 overexpression, followed by analysis of placental tissue for relevant protein expression and signaling pathway activation.

**Results:**

H/R treatment induced autophagy in HUVECs, as evidenced by increased LC3-II/LC3-I ratio and decreased p62 levels. It also triggered ferroptosis, characterized by elevated iron, ROS, and MDA levels, along with reduced GSH and GPX4 expression. Overexpression of SIRT3 suppressed both H/R-induced autophagy and ferroptosis, and these protective effects were reversed by the autophagy inducer rapamycin or the ferroptosis inducer erastin. Mechanistically, SIRT3 activated the PI3K/Akt/mTOR signaling pathway, and inhibition of this pathway attenuated the suppressive effects of SIRT3 on autophagy and ferroptosis. In PIH rats, SIRT3 overexpression increased the levels of SIRT3 and GPX4, enhanced phosphorylation of PI3K, Akt, and mTOR, and reduced the LC3-II/LC3-I ratio in placental tissues compared to control PIH rats.

**Discussion:**

These findings demonstrate that SIRT3 protects against hypoxia-induced endothelial cell injury by inhibiting autophagy-dependent ferroptosis through activation of the PI3K/Akt/mTOR signaling pathway. The in vivo results further support a protective role of SIRT3 in alleviating PIH. This study reveals a novel SIRT3-mediated regulatory mechanism in PIH pathophysiology, suggesting that targeting SIRT3 and its downstream signaling may represent a potential therapeutic strategy for the management of PIH.

## 1 Introduction

Pregnancy-induced hypertension (PIH) refers to a group of diseases characterized by elevated blood pressure during pregnancy and affects approximately 7–15% of all pregnancies (Marek et al., 2021; Shrestha et al., 2021). This condition seriously affects the health of pregnant women, postpartum women, and newborns and is a major cause of increased maternal and perinatal morbidity and mortality (Wei et al., 2022). It is also a multisystemic functional disorder, primarily characterized by hypertension, edema, and proteinuria. Typically, PIH manifests after 20 weeks of gestation, and symptoms resolve after delivery. The incidence of PIH has gradually increased over time, which increases the risk of maternal and perinatal mortality (American College of Obstetricians and Gynecologists, 2020). At present, the etiology and pathogenesis of PIH remain unclear. Multiple factors and pathways can lead to the occurrence of hypertensive disorder that complicates pregnancy. The activation and injury of the diffuse vascular endothelial cells of the maternal body are an important reason for the pathogenesis of PIH, which leads to various clinical symptoms, such as hypertension, proteinuria, systemic inflammatory response, and accumulation of anti-angiogenic factors (Wu et al., 2021; Zhu L. et al., 2023). However, the mechanism of endothelial cell activation and injury is also unclear. Assessment of the severity of PIH often involves the detection of vascular endothelial cell damage, which is crucial for its clinical prevention, diagnosis, and treatment.

Ferroptosis is a new form of programmed cell death that unaffected by the commonly used caspase inhibitors, which is in contrast to other types of programmed cell death. This mode of cell death is characterized by the disruption of the balance between lipid peroxidation and antioxidant defense and results in iron-dependent lipid hydroperoxide accumulation and overproduction of reactive oxygen species (ROS). These changes cause cell membrane damage and rupture, which result in the leakage of cell contents (Jiang et al., 2021; Ma et al., 2022). The main morphological manifestations of ferroptotic cells include the loss of plasma membrane integrity, substantial swelling of organelles and cytoplasm, and massive chromatin condensation (Park et al., 2025). The impaired lipid metabolism of the cell membrane causes the massive accumulation of lipid peroxides, which exceed the reduction limit of glutathione peroxidase (GPX); the ferrous-ion Fenton reaction then catalyzes the generation of ROS or lipid free radicals, and their massive accumulation leads to cell death (Seibt et al., 2024). DJ-1 plays a protective role in pre-eclampsia by inhibiting ferroptosis in trophoblast cells through upregulation of the nuclear factor erythroid 2-related factor 2/GPX4 signaling pathway (Liao et al., 2022).

Sirtuins (SIRT) belong to class III histone deacetylases, which are widely distributed in the human body and participate in the management of cellular stress response, metabolic regulation, aging, and apoptosis. SIRT3 i = s a gene located in the p15.5 region of chromosome 11, which is mainly localized in the mitochondria. SIRT3 regulates mitochondrial metabolism and oxidative stress and is widely distributed in human normal tissues, especially in skeletal muscle, liver, kidney, and cardiac muscle, where mitochondria are abundant. SIRT3 is initially expressed in the cytoplasm (Dikalov and Dikalova, 2022; Nahálková, 2022), and its inactive precursor possesses an N-terminal mitochondrial localization signal peptide. When cells are stressed, SIRT3 enters the mitochondria and is captured by the mitochondrial matrix processing peptidase, which processes it into a short active form of SIRT3 that comprises 257 amino acid residues. In oxidative stress, the body generates ROS and induces cytotoxicity under the stimulation of some factors. ROS are mainly produced during redox reaction and highly reactive oxygen-containing substances. Ferroptosis is a cell death mode that involves oxidative stress and inflammation and contributes to the occurrence and progression of a variety of diseases.

In sepsis-induced myocardial injury, SIRT3 considerably attenuated LPS-induced H9C2 cell death, inflammation, oxidative stress, and ferroptosis (Qin et al., 2023). Resveratrol reduced ROS-induced ferroptosis by activating SIRT3 and compensating the glutathione (GSH)/GPX4 pathway, which alleviated intestinal ischemia–reperfusion injury (Wang et al., 2023). In the progression of pre-eclampsia, the decrease in SIRT3 indicates that the placenta lacks reserves of metabolic energy efficiency, increases inflammation, and decreases resistance to environmental stressors (Broady et al., 2017). The loss of SIRT3 may interfere with the normal functions of trophoblast cells, including their ability to move and invade surrounding tissues, which hinders the formation of fallopian tubes and promotes cell necrosis. This phenomenon is related to the occurrence of preeclampsia, a pregnancy complication (Yu et al., 2022). Focusing on the role of SIRT3 in the regulation of oxidative stress in various diseases, we determined whether SIR3 contributes to the pathogenesis of PIH by affecting ferroptosis. Related reports are lacking, and this topic must be further explored.

## 2 Materials and methods

### 2.1 Cell culture and clinical samples

Human umbilical vein endothelial cells (HUVECs, PCS-100-013^™^) were purchased from ATCC (Manassas) and cultured in endothelial-cell basal medium supplemented with 10% fetal bovine serum, 10 mM HEPES (Sigma Aldrich), and 1% penicillin/streptomycin solution in a humidified atmosphere containing 5% CO_2_. Thirty cases of hospitalized patients in the Department of Obstetrics and Gynecology of our hospital from 2021 to 2023, including 15 cases of normal term pregnancy cesarean section and 15 cases of PIH cesarean section (7 cases of gestational hypertension, 5 cases of pre-eclampsia, 3 cases of severe eclampsia), were collected. After delivery of the placenta, the tissues (1 cm^3^) on its maternal surface were immediately removed from the central zone for paraffin embedding. Several 1 cm^3^ placental tissues were collected and stored at −80 °C for subsequent studies. This study received approval from the Ethics Committee of Shaanxi Provincial People’s Hospital and conformed to the provisions of the Declaration of Helsinki. Placental tissue samples were collected at Shaanxi Provincial People’s Hospital, and all participants agreed and signed the informed consent prior collection.

### 2.2 Cell transfection

pcDNA-SIRT3 and vector were purchased from RiboBio Co., Ltd. (Guangzhou, China). Cell transfection was carried out when the HUVECs grew to 80% confluence. Sterile 1.5 mL centrifuge tubes without enzymes were prepared and marked based on the grouping. The cells were added with 2 μL pcDNA3.1(+)-SIRT3 plasmid or vector and 100 μL Opti-MEM in a six-well plate. Lipofectamine^™^ 2000 and Opti-MEM without serum were also added at the same concentration to the centrifuge tube of the same group. After standing for 5 min, the pcDNA3.1(+)-SIRT3 plasmid was mixed with Lipofectamine^™^ 2000 and incubated at room temperature for 20 min. All the media in the six-well plate was removed using a Pasteur pipette, and the sample was added with 200 μL plasmid and transfection reagent mixture. The plate was stirred gently and placed in an incubator for culture. Cell state was observed at 6 h after transfection, and cells were collected 24 h after transfection for subsequent studies. Rapamycin was purchased from Sigma-Aldrich (catalog number: R0395). For the preparation of the working solution, rapamycin powder was first dissolved in a small volume of dimethyl sulfoxide and then diluted with culture medium to a final concentration of 10 nM. HUVECs were treated with 10 nM rapamycin for 24 h, a concentration selected based on previous studies, to induce autophagy (Niu et al., 2019), and preliminary experiments were conducted in our laboratory.

### 2.3 Animals

Female Sprague–Dawley (SD) rats (5 weeks old, weighing 230 g, Wuhan Experimental Animal Center) were housed in a SPF environment with a constant temperature of 25 °C and a 12/12 h light/dark cycle and fed with standard rat feed and tap water. Pregnancy was induced by housing two female rats together with a weight-matched male rat. The appearance of vaginal plugs on the day after mating indicates the beginning of pregnancy, that is, gestational day (GD) 1. Pregnant SD rats were randomly divided into four groups, with three rats in each group: control, PIH, PIH + vector group, and PIH + pcDNA-SIRT3 groups. In the PIH group, L-NAME (Sigma, St Louis, MO, United States) was added to the drinking water at 50 mg/kg/day from GD8 until GD19. Pregnant rats in the control group drank water normally. For the SIRT3 overexpression group, 500 μL SIRT3 plasmid–liposome complex was intraperitoneally injected into pregnant mice once every 3 days. The negative control group was injected with the same amount of vector–liposome complex. Systolic blood pressure and 24 h urinary protein content were measured at GD12, GD16, and GD20. The rats were euthanized by isoflurane at GD20, and placental tissue samples were collected. All animal experiments were approved by the animal care and use committee of XX and conducted in accordance with the National Institutes of Health Animal Welfare guidelines.

### 2.4 Western blot analysis

Proteins from cells and tissues were extracted using radioimmunoprecipitation assay lysis buffer, separated by sodium dodecyl sulfate–polyacrylamide gel electrophoresis, and then transferred onto polyvinylidene fluoride membranes. Protein samples were collected from three independent cell cultures per treatment group, or proteins were extracted from the placental tissues of three individual mice per group. The membrane was blocked in skimmed milk powder at room temperature for 2 h and then incubated with the following primary antibodies overnight at 4 °C: GAPDH (1:1,500), light chain 3 (LC3) B (1:2,000), sequestosome 1 (SQSTM1)/p62 (1:10,000), SIRT3 (1:500), GPX4 (1:2,000), phosphoinositide 3-kinase (PI3K, 1:1,000), PI3K (phospho Y467, 1:100), protein kinase B (Akt) (1:500), Akt (phospho T308, 1:1,000), mechanistic target of rapamycin (mTOR) (1:2,000), and mTOR (phospho S2448, 1:1,500). The membrane was then incubated with goat anti-rabbit IgG antibody conjugated with horseradish peroxidase for 60 min at room temperature. Enhanced chemiluminescence detection reagents (Bethesda, MD, United States) were used to visualize protein bands.

### 2.5 Immunohistochemical assay

The embedded block of placental tissue was frozen at −20 °C for 30 min and immediately sliced at 5 μm thickness. The slides with the placental tissue were placed on an 80 °C toaster for 45 min and hydrated with graded ethanol (100, 95, 90, 80, and 75%) after passing through the second nail. After the sodium citrate buffer was heated to boil, the paraffin sections were placed in the buffer, heated, and naturally cooled for 30 min. The sections were washed with phosphate-buffered saline (PBS), and a circle was drawn around the tissue using an immunohistochemical pen. Each circle was added with 100 μL 5% BSA and blocked at room temperature for 20 min. The blocking solution was shaken off, and the sections were rapidly added with the pre-prepared SIRT3 and GPX4 antibodies dropwise and incubated overnight at 4 °C. The sections were incubated again at 37 °C for 45 min and washed thrice with PBS buffer. The sections were added with 50 μL solution at room temperature and incubated for 30 min. They were then rinsed with PBS buffer again, dried, and tiled in a wet box. Finally, 50 μL 3,3′-diaminodbenzidine solution at working concentration was added to the sections for color reaction. After incubation for 30 s at 37 °C, color development was terminated with distilled water for 10 min. The sections were counterstained with hematoxylin for 2 min, washed with water for 10 min, dehydrated with graded ethanol, and sealed with neutral gum. Images were acquired using a bio intelligent image Navigator (Olympus FSX-100), and quantitative analysis was performed using Image-Pro Plus software.

### 2.6 Cell Counting Kit-8 assay

Single-cell suspensions were prepared through trypsin treatment of cells in the logarithmic growth phase. The cells were seeded in 96-well plates at a density of 1 × 10^4^ cells/well and cultured in a constant-temperature incubator for 24 h. Cells in each well were added with 10 μL Cell Counting Kit-8 (CCK-8) reagent and incubated for 4 h. Optical density was recorded at 450 nm via enzyme-linked immunosorbent assay.

### 2.7 GSH content detection

The cells were digested with 0.25% trypsin for 5 min and centrifuged at 5,000 rpm for 10 min to obtain the precipitate. Then, the cells were resuspended in PBS, sonicated, and centrifuged for 10 min to collect the supernatant. In accordance with the guidelines provided by the manufacturer, absorbance was recorded and used to calculate the GSH content.

### 2.8 GSH-Px activity assay

The placental tissue was ground into homogenate and centrifuged at 3,500 rpm for 10 min. The supernatant was collected and used for recording of absorbance. The activity of GSH-Px was determined following the guidelines provided by the manufacturer.

### 2.9 Iron content detection

The total iron content in cells and tissues was determined using the corresponding detection kits. No less than 3 × 10^6^ cells were harvested from the culture bottle for analysis, and 300 μL extraction solution was used to lyse the cells. Based on the grouping, the centrifuge tubes were divided into blank, standard, and sample tubes for testing. About 20 μL distilled water was added to the blank tube, 20 μL standard substance to the standard tube, and 20 μL sample to the test tube. Each tube was then added with 180 μL of the mixture of reagents I, II, and III and mixed. The liquid in the centrifuge tube was transferred to the enzyme plate and left at room temperature for 10 min. Absorbance at 510 nm was detected via spectrophotometry.

### 2.10 Malondialdehyde content detection

The malondialdehyde (MDA) levels in cells and tissues were detected using MDA assay kits. No less than 3 × 10^6^ cells were harvested from the culture flasks and lysed with the extraction solution. Based on the grouping, 100 μL absolute ethanol, standard, and sample were added to the blank, standard, and sample tubes, respectively. Each tube was added with 1 mL working solution, placed in a water bath at 100 °C for 40 min, and centrifuged at 1,000 × g for 10 min to remove insoluble substances. The liquid in the centrifuge tube was transferred to the microplate and incubated at 37 °C in the dark for 1 h. Absorbance at 532 nm was recorded, and MDA content was calculated in accordance with the manufacturer’s instructions.

### 2.11 Cellular immunofluorescence

The cell suspension was placed in the cell seed plate. When the cell density reached 60% and the cells showed even distribution, a pipette gun was used to aspirate the medium. The cells were washed with PBS for 10 min, added with 4% paraformaldehyde, fixed to the climbing piece at room temperature for 15 min, and then washed again with PBS thrice for 10 min each time. The cells were added with 0.2% Triton X-100 solution and washed thrice with PBS for 5 min after permeabilization at room temperature. The cells were dropwise added with phalloidin antibody diluent (1:100), incubated in the dark at room temperature for 20 min, and rinsed three times with PBS for 5 min each time. Finally, the cells were added with 100 ng/mL 4′,6-diamidino-2-phenylindole solution dropwise, incubated at room temperature in the dark for 5 min, and rinsed with PBS three times, for 5 min each time. A clean slide was prepared and added with an antiquenching sealing tablet. The climbing slide was buckled upside down and dropped with nail polish to fix it before being placed in the cassette. After the climbing piece was wiped dry, image was collected using an inverted fluorescence microscope.

### 2.12 ROS level detection

ROS content in cells and tissues was detected using ROS detection kits. The 2′,7′-dichlorodihydrofluorescein diacetate probe was added to the treated cells at a ratio of 1:1,000 and incubated in a water bath at 37 °C for 30 min. The residual probe was purged with PBS and centrifuged at 1,000 rpm for 5 min. The cells were recultured with PBS and transferred onto a 96-well plate. An inverted fluorescence microscope (488 nm excitation wavelength, 525 nm emission wavelength) was used for observation, and Image J was used for analysis.

### 2.13 Reverse transcription-quantitative polymerase chain reaction

We used the Trizol method (Invitrogen, United States) in the extraction of middle RNA. cDNA was prepared using relevant reagent kits (Takara). Reverse transcription-quantitative polymerase chain reaction (RT-qPCR) was performed using SYBR green (Takara, China). The primers (Sangon) were as follows: SIRT3 forward, 5′-CCC TGG AAA CTA CAA GCC CAA C-3′, reverse, 5′-GCA GAG GCA AAG GTT CCA TGA G-3′; GAPDH, 5′-GTC TCC TCT GAC TTC AAC AGC G-3′, reverse, 5′-ACC ACC CTG TTG CTG TAG CCA A-3′. Normalized relative expression level was determined using 2^−ΔΔCt^ method.

### 2.14 Statistical analysis

All data were analyzed by SPSS software (version 22.0). Quantitative data from three independent experiments were presented as mean ± standard deviation (mean ± SD). Comparisons between multiple groups were conducted using analysis of variance with a least-significant difference (LSD) post-hoc analysis (*N* = 3). Differences at *p* < 0.05 indicated statistical significance.

## 3 Results

### 3.1 Ferroptosis marker levels were increased in PIH placental tissues

**Figure 1:**
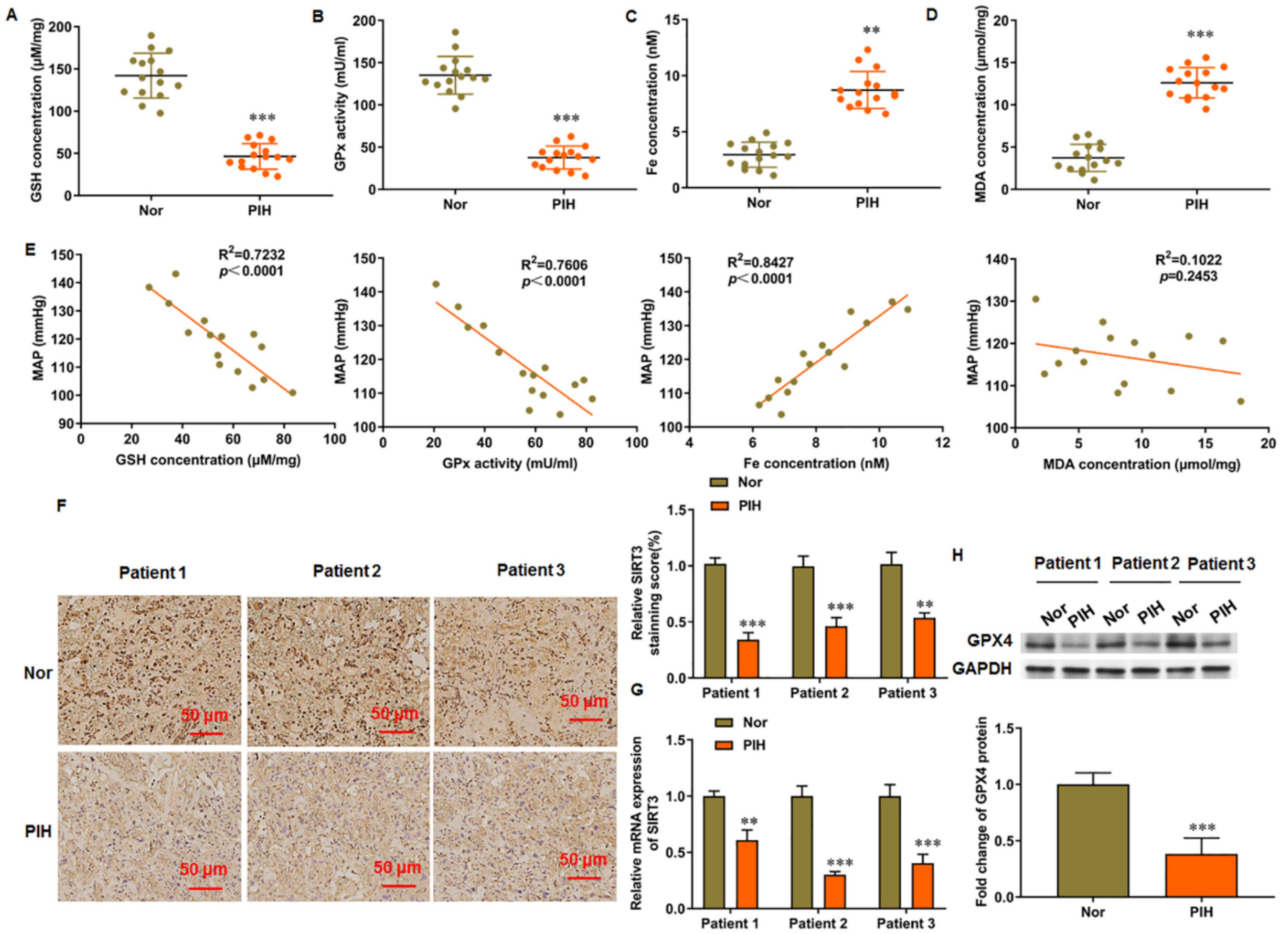
Levels of ferroptosis related genes in placenta of patients with PIH. Placental tissues were collected from patients with PIH (*N* = 15) and physiologically normal pregnancies subjects (*N* = 15). **(A)** GSH content. **(B)** GSH-Px activity. **(C)** Total Fe (II) content. **(D)** MDA level. **(E)** Correlation analysis between maternal mean arterial pressure (MAP) and levels of ferroptosis markers in patients with PIH. **(F)** Representative images of immunohistochemistry of SIRT3 in placental tissue. Scale bar = 50 μm. **(G)** SIRT3 level in placental tissue was detected by RT-qPCR. **(H)** Protein levels of GPX4 was detected by Western blot analysis. Placental samples were obtained from patients with PIH (*N* = 3) and subjects with physiologically normal pregnancy (*N* = 3). ^**^p < 0.01 and ^***^p < 0.001.

Placental tissues were collected from normal pregnant women and patients with PIH after delivery and used for detection of ferroptosis-related indicators. The GSH content (Figure 1A) and activity of GSH-Px (Figure 1B) were decreased, whereas the content of free iron (Figure 1C) and levels of MDA (Figure 1D) were increased in the placental tissue of patients with PIH after delivery. Correlation analysis was conducted between the maternal mean arterial pressure (MAP) of the patients and the above related indicators. MAP decreased with the increase in GSH content and GSH-Px activity and exhibited a positive correlation with free iron content but not with MDA level in the placenta (Figure 1E). Immunohistochemistry and RT-qPCR results indicate the increased SIRT3 levels in the placental tissues (Figures 1F,G, respectively). In addition, GPX4 levels were decreased in the placental tissues of patients with PIH (Figure 1H). These results suggest that ferroptosis is accompanied with the progression of PIH, which may be related to the dysregulation of SIRT3 levels.

### 3.2 SIRT3 inhibits autophagy in hypoxia/reoxygenation-treated HUVECs

**Figure 2:**
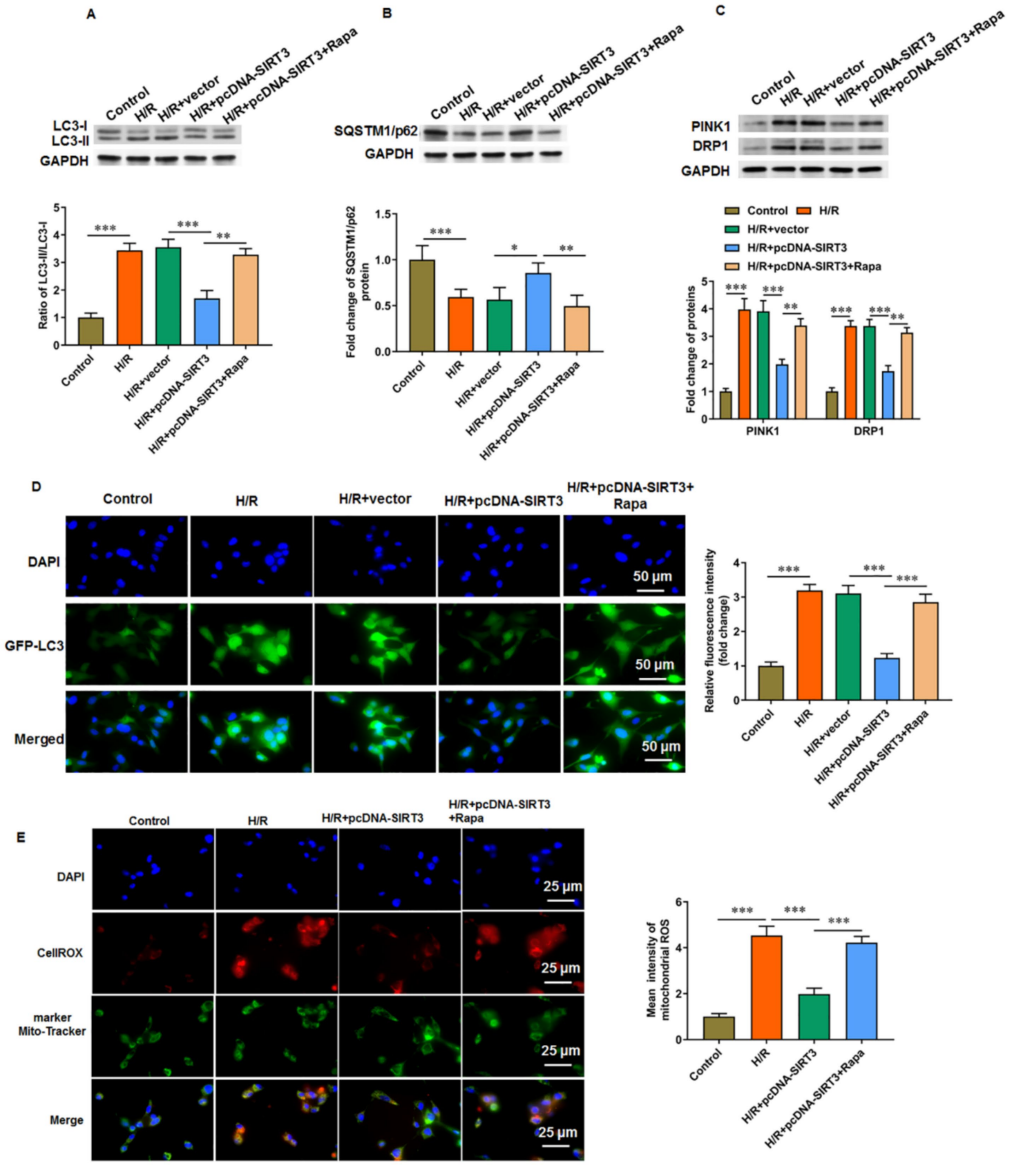
SIRT3 inhibits H/R-induced autophagy in HUVECs. H/R-treated HUVECs were transfected with pcDNA-SIRT3 alone or co-treated with rapamycin (Rapa). **(A)** Protein levels of LC-3II and LC-3I. **(B)** Protein levels of SQSTM1/p62. **(C)** Protein levels of PINK1 and DRP1. **(D)** Representative images of immunofluorescence of autophagy specific protein LC-3II. Scale bar = 50 μm. **(E)** Mitochondrial ROS levels were evaluated by CO-staining of mitochondrial marker Mito tracker and cellrox (a ROS indicator). Scale bar = 25 μm. Rapamycin was used to activate autophagy (*N* = 3). ^*^p < 0.05, ^**^p < 0.01, and ^***^p < 0.001.

After hypoxia/reoxygenation (H/R) treatment, the ratio of LC3-II/LC3-I in HUVECs was increased, and the level of SQSTM1/p62 was decreased. These findings suggest that H/R treatment induced autophagy in HUVECs. We then transfected pcDNA-SIRT3 into H/R-treated HUVECs and observed that SIRT3 inhibited the autophagy induced by H/R treatment. Rapamycin primarily acts as an mTOR inhibitor, particularly by inhibiting mTOR complex 1 (mTORC1), which relieves the suppression of the autophagy process and effectively promotes autophagy activation. Under the action of rapamycin, the ratio of LC-3 II/LC-3 I in HUVECs was increased (Figure 2A), and the level of SQSTM1/p62 was decreased (Figure 2B). When cells experience hypoxia followed by re-exposure to normal oxygen levels, a series of complex cellular responses is triggered, including increased autophagy and mitophagy, in which key regulatory proteins PTEN-induced putative kinase 1 (PINK1) and dynamin-related protein 1 (DRP1) are involved in the recognition of damaged mitochondria and their subsequent clearance. We observed that H/R treatment increased the levels of PINK1 and DRP1. Overexpression of SIRT3 suppressed the levels of PINK1 and DRP1, whereas re-activation of autophagy by Rapa led to subsequent increases in PINK1 and DRP1 levels (Figure 2C). Similarly, immunofluorescence analysis showed that the green fluorescence signal of LC-3 II, a marker protein of autophagy, was enhanced in the H/R group compared with that in the control group. The signal decreased in H/R-treated HUVECs transfected with pcDNA-SIRT3, but rapamycin promoted again the levels of autophagy protein LC-3 I (Figure 2D). In addition, we measured mitochondrial ROS levels and observed that H/R treatment increased mitochondrial ROS production. Overexpression of SIRT3 reduced the mitochondrial ROS levels, but ROS levels were increased again upon autophagy activation (Figure 2E).

### 3.3 SIRT3 inhibits ferroptosis in H/R-treated HUVECs

**Figure 3:**
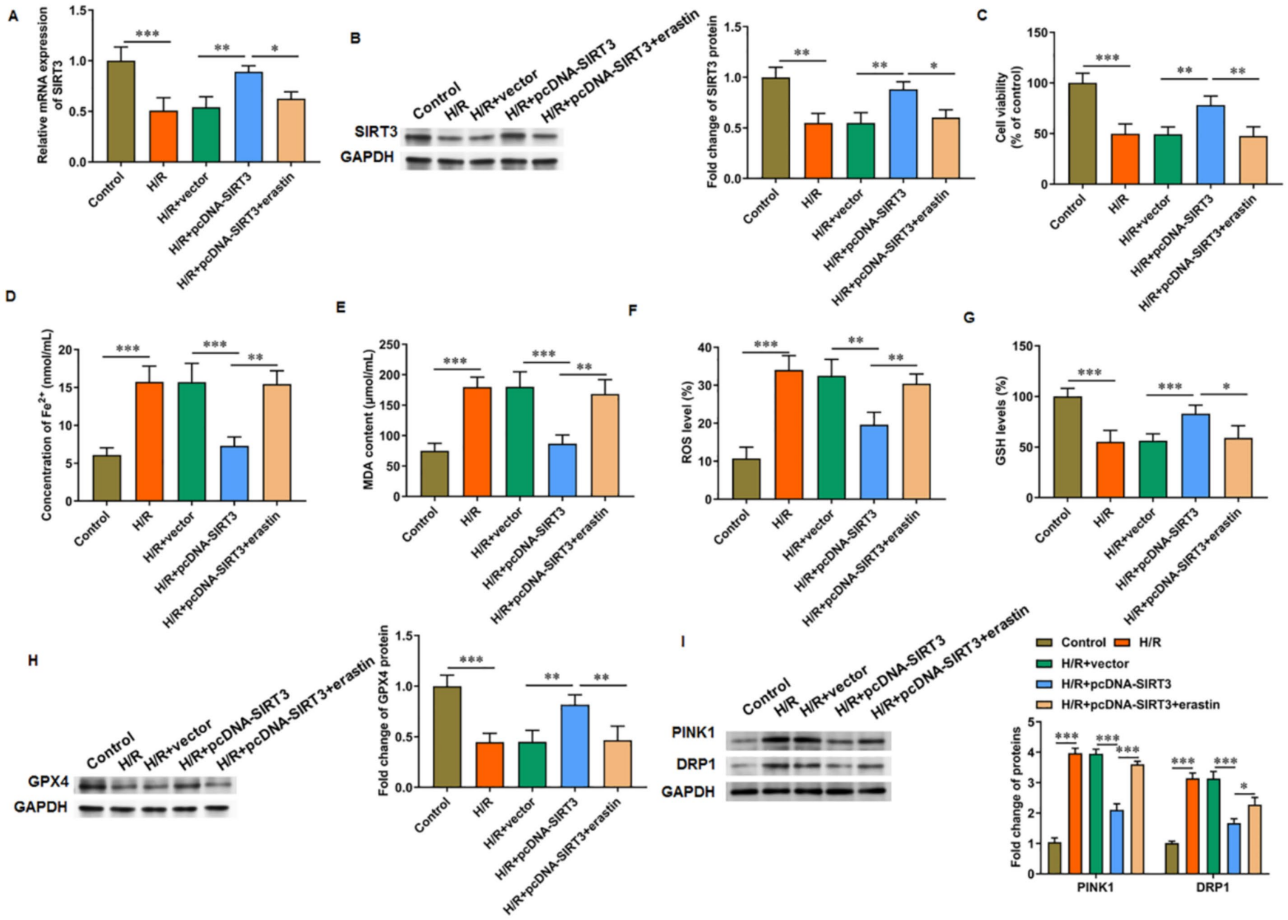
SIRT3 inhibits H/R-induced ferroptosis in HUVECs. H/R-treated HUVECs were transfected with pcDNA-SIRT3 alone or co-treated with erastin. **(A,B)** The mRNA and protein levels of SIRT3. **(C)** Cell viability. **(D)** Fe^2+^ content. **(E)** MDA content. **(F)** ROS levels. **(G)** GSH content. **(H)** Protein levels of GPX4. **(I)** Protein levels of PINK1 and DRP1. Erastin was used to activate ferroptosis (*N* = 3). ^*^p < 0.05, ^**^p < 0.01, and ^***^p < 0.001.

Compared with normal cultured cells, H/R-treated HUVECs showed decreased SIRT3 mRNA (Figure 3A) and protein (Figure 3B) levels, reduced cell viability (Figure 3C), and increased iron ion content (Figure 3D), MDA content (Figure 3E), and ROS level (Figure 3F). In addition, the GSH level (Figure 3G) and GPX4 protein levels were decreased (Figure 3H) in H/R-treated HUVECs. The pcDNA-SIRT3 plasmid was then transfected to H/R-treated HUVECs. SIRT3 overexpression reversed the H/R-induced ferroptosis in HUVECs. However, under the action of erastin, a ferroptosis agonist, the inhibitory effect of SIRT3 this outcome was abolished. Furthermore, the overexpression of SIRT3 reversed the H/R-induced increase in the levels of PINK1 and DRP1 proteins. However, upon erastin-induced ferroptosis activation, the levels of PINK1 and DRP1 were increased, which suggests that mitochondrial autophagic damage accompanied ferroptosis (Figure 3I). These results show that SIRT3 can inhibit the ferroptosis of H/R-treated HUVECs.

### 3.4 SIRT3 inhibits autophagy-dependent ferroptosis in H/R-treated HUVECs

**Figure 4:**
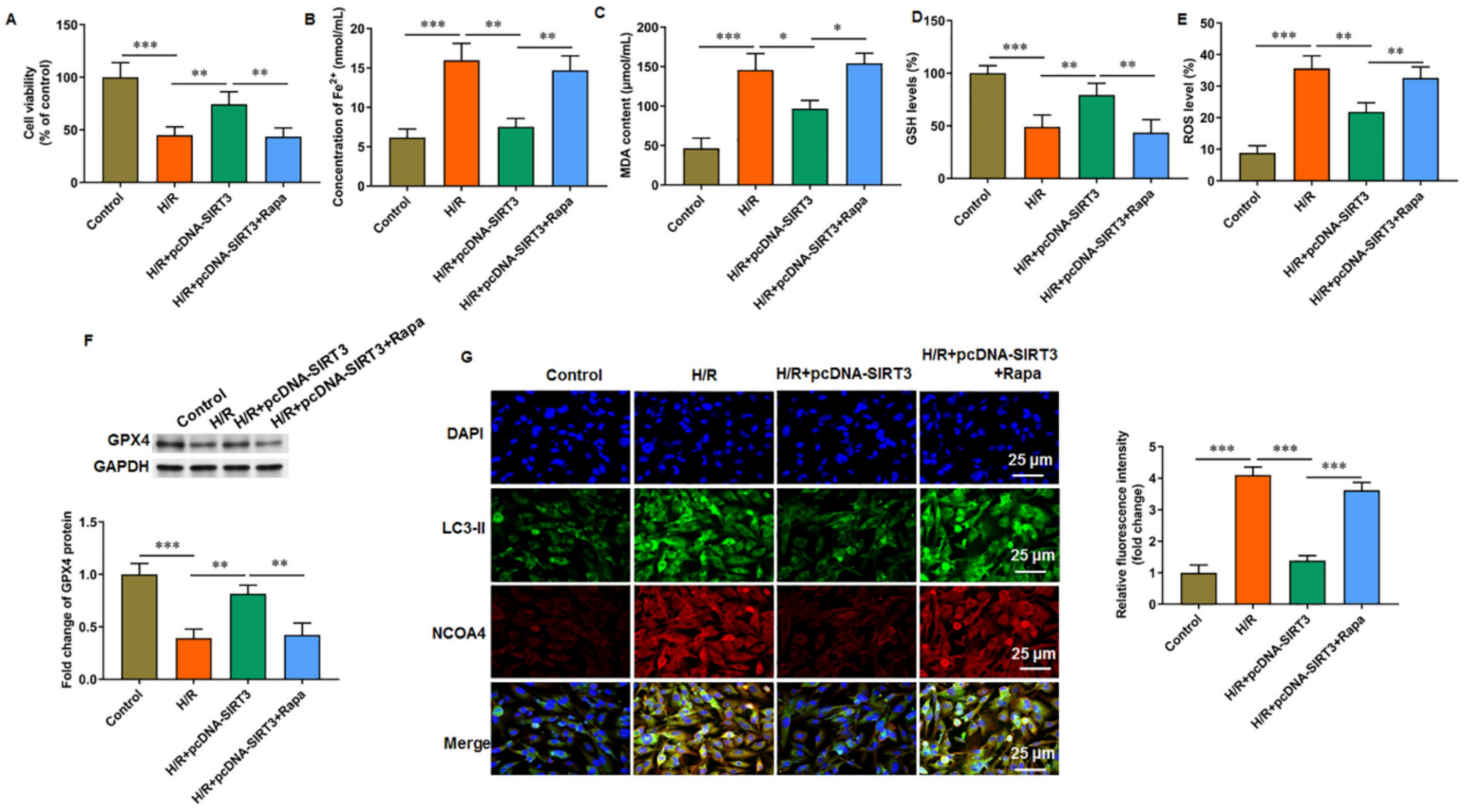
SIRT3 inhibits H/R-induced autophagy-dependent ferroptosis in HUVECs. H/R-treated HUVECs were transfected with pcDNA-SIRT3 alone or co-treated with rapamycin (Rapa). **(A)** Cell viability. **(B)** Fe^2+^ content. **(C)** MDA content. **(D)** GSH content. **(E)** ROS levels. **(F)** Protein levels of GPX4. **(G)** Representative images of immunofluorescence of LC-3II colocalized with NCOA4. Erastin was used to activate ferroptosis. Nuclear receptor coenzyme activating factor 4 (NCOA4) is a key receptor for iron autophagy. Rapamycin was used to activate autophagy. Scale bar = 25 μm (*N* = 3). ^*^p < 0.05, ^**^p < 0.01, and ^***^p < 0.001.

Rapamycin was used to activate autophagy to evaluate its involvement in SIRT3-mediated ferroptosis. For H/R-treated HUVECs with SIRT3 overexpression, cell viability (Figure 4A), GSH level (Figure 4D), and GPX4 protein levels (Figure 4F) were increased, and iron ion content (Figure 4B), MDA content (Figure 4C), and ROS level were decreased (Figure 4E). After rapamycin treatment, the inhibitory effect of SIRT3 on H/R-induced ferroptosis was reversed. As a key receptor of iron autophagy, nuclear receptor coenzyme activator 4 (NCOA4) and LC-3 II colocalized in large amounts in H/R-treated HUVECs. The occurrence of ferroptosis depends on autophagy, with SIRT3 inhibiting autophagy-dependent ferroptosis. In the rapamycin-treated group, large amounts of NCOA4 and LC-3 II aggregation were observed (Figure 4G).

### 3.5 SIRT3 inhibits ferroptosis and autophagy by activating the PI3K/Akt/mTOR pathway

**Figure 5:**
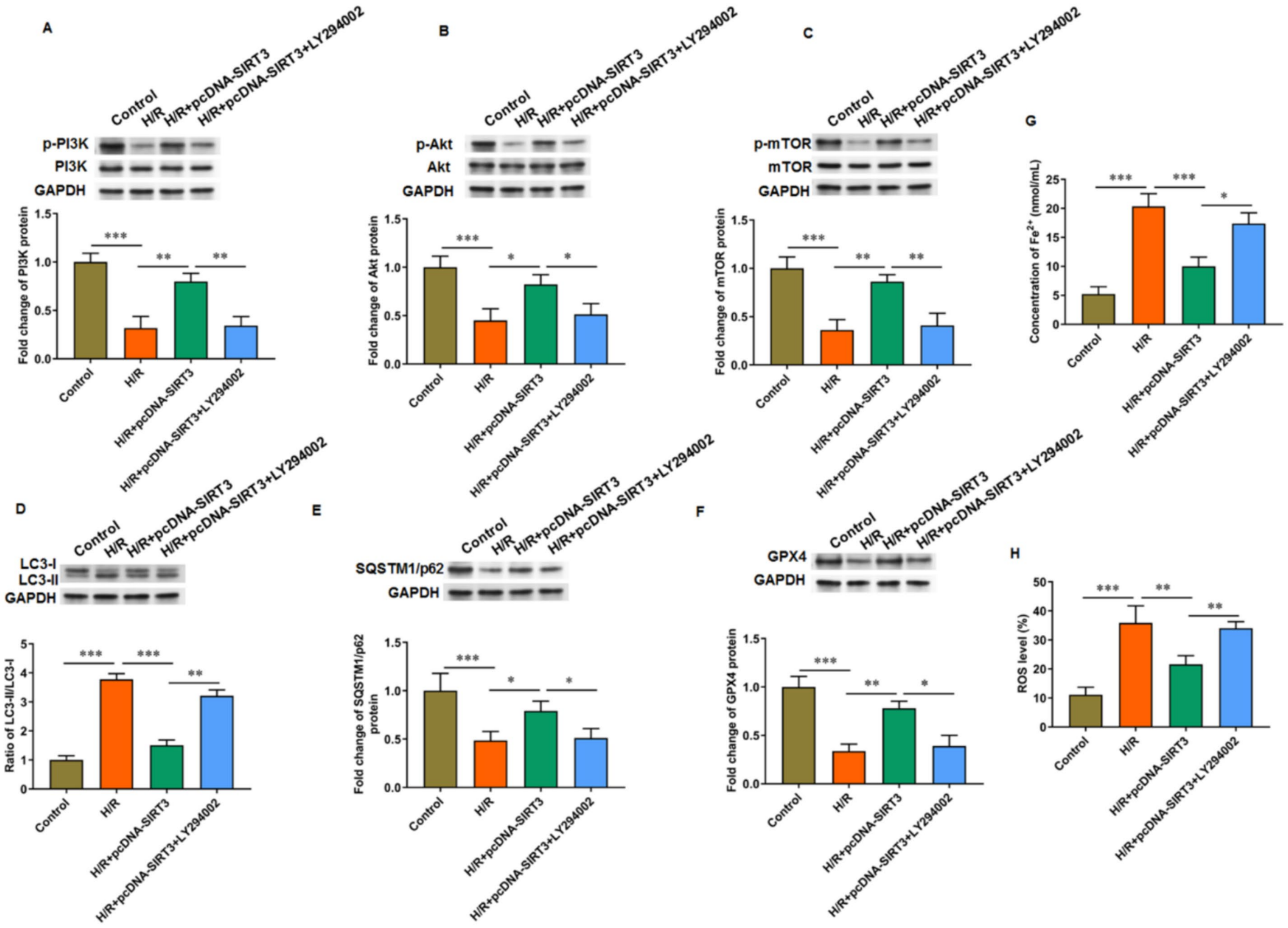
SIRT3 inhibits H/R-induced autophagy and ferroptosis in HUVECs by activating PI3K/Akt/mTOR pathway. H/R-treated HUVECs were transfected with pcDNA-SIRT3 alone or co-treated with LY294002. **(A–C)** Phosphorylation levels of PI3K, Akt, and mTOR proteins. **(D)** Protein levels of LC-3II and LC-3I. **(E)** Protein levels of SQSTM1/p62. **(F)** Protein levels of GPX4. **(G)** Fe^2+^ content. **(H)** ROS levels. LY294002 was used to inhibit PI3K activation (*N* = 3). ^*^p < 0.05, ^**^p < 0.01, and ^***^p < 0.001.

LY294002 was used to inhibit PI3K activation to explore the role of the PI3K/Akt/mTOR signaling pathway in SIRT3-regulated ferroptosis. In HUVECs subjected to H/R treatment, the phosphorylation levels of PI3K, Akt, and mTOR proteins were decreased. Treatment with SIRT3 reversed the inhibitory effect of the PI3K/Akt/mTOR pathway caused by H/R. After the intervention with LY294002, the phosphorylation levels of PI3K, Akt, and mTOR proteins were decreased again (Figures 5A–C). When LY294002 inhibited PI3K activation, the ratio of LC-3 II/LC-3 I was increased (Figure 5D), the protein levels of SQSTM1/p62 (Figure 5E) and GPX4 (Figure 5F) were decreased, and the levels of iron ion (Figure 5G) and ROS (Figure 5H) were increased in H/R-treated HUVECs overexpressing SIRT3. The results confirm that SIRT3 inhibited ferroptosis and autophagy in H/R treated HUVECs through activation of the PI3K/Akt/mTOR pathway.

### 3.6 SIRT3 alleviates PIH progression in rats

**Figure 6:**
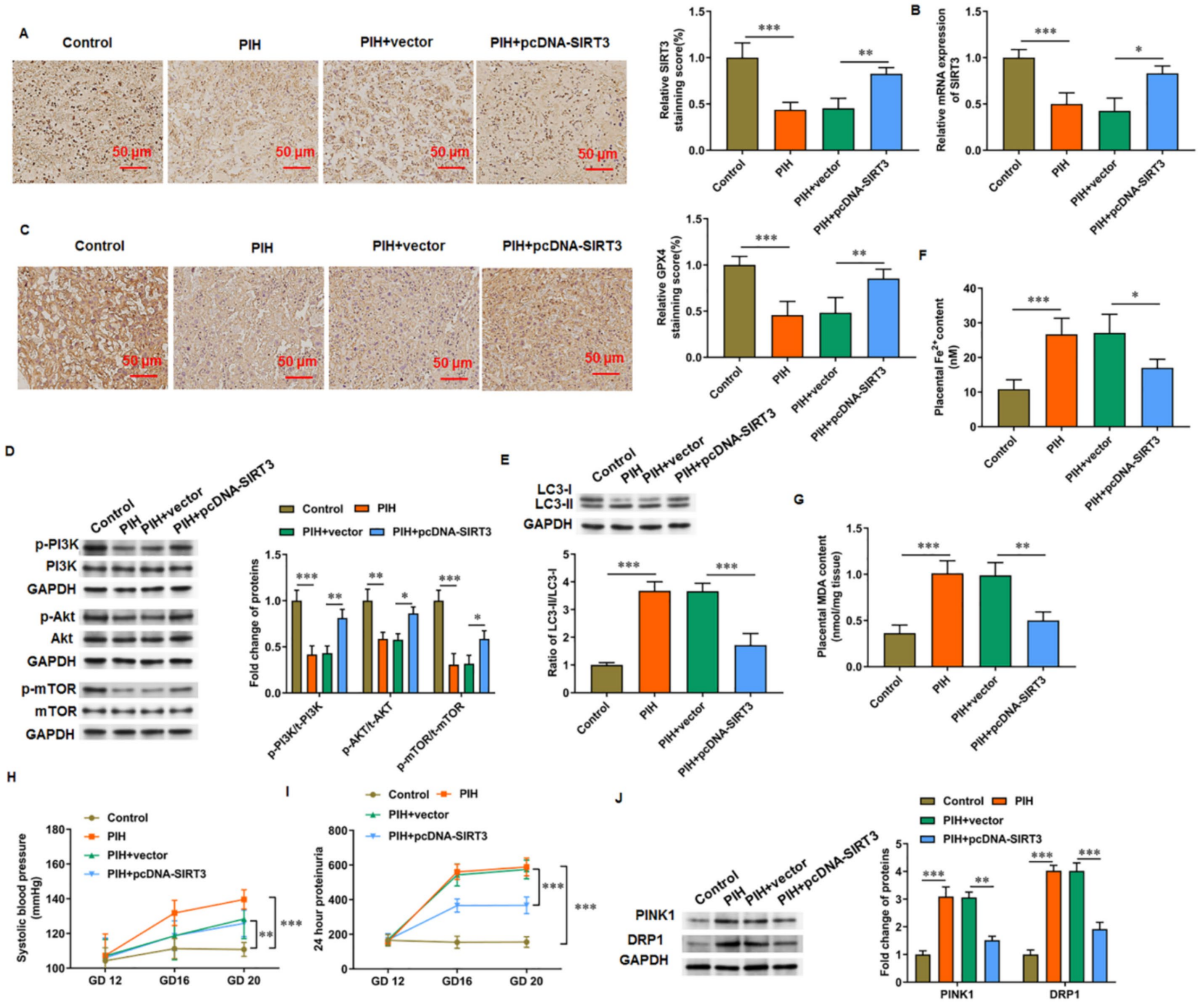
SIRT3 delays PIH progression in rats. **(A,B)** SIRT3 levels was detected by immunohistochemistry and RT-qPCR. **(C)** Representative immunohistochemical images of GPX4. Scale bar = 50 μm. **(D)** Phosphorylation levels of PI3K, Akt, and mTOR proteins. **(E)** Protein levels of LC-3II and LC-3I. **(F)** Fe^2+^ content. **(G)** MDA content. **(H)** Systolic blood pressure. **(I)** 24-h urinary protein content. **(J)** Protein levels of PINK1 and DRP1 (*N* = 3). ^*^p < 0.05, ^**^p < 0.01, and ^***^p < 0.001.

The levels of SIRT3 (Figures 6A,B) and GPX4 (Figure 6C) in the placental tissue were decreased in PIH rats but increased in PIH rats overexpressing SIRT3. The phosphorylation levels of PI3K, Akt, and mTOR proteins were increased in the placenta of SIRT3 overexpressing rats compared with those in PIH rats (Figure 6D). The ratio of LC-3 II/LC-3 I in the placental tissue of PIH rats was increased, which indicates that PIH progression was accompanied with abnormal autophagy and mediated by SIRT3 loss. When PIH rats overexpressed SIRT3, the ratio of LC-3 II/LC-3 I in the placental tissue was decreased (Figure 6E). In addition, the progress of PIH was accompanied with ferroptosis, which was manifested by the increase in iron ion (Figure 6F) and MDA (Figure 6G) content in the placenta of PIH rats. Meanwhile, SIRT3 inhibited the production of Fe^2+^ and MDA. Systolic blood pressure and 24 h urinary protein are important indicators used to evaluate PIH, and they showed decreases (Figures 6H,I, respectively) in PIH rats with SIRT3 overexpression compared with those without. Similarly, in placental tissues from PIH rats, the protein levels of PINK1 and DRP1 were increased, and overexpression of SIRT3 reduced their levels (Figure 6J).

## 4 Discussion

Autophagy represents an evolutionarily conserved life process in all eukaryotic cells. It wraps proteins, organelles, and other substrates in cells through a double-layer membrane structure to form autophagic vesicles. Under the action of a microtubule system, autophagic vesicles fuse with lysosomes, and their contents are degraded into monosaccharides, amino acids, and fatty acids under the action of lysosomal enzymes, which are reused by cells (Liu et al., 2023). Autophagy plays an important role in maintaining cell homeostasis and other biological processes, such as cell survival, cell differentiation, development, immunity, and removal of harmful macromolecules, under stress and nutrient deficiency conditions (Klionsky et al., 2021). This study revealed that LC3-II levels in the placental tissue of patients with severe preeclampsia was increased compared with those in normal pregnant women. In addition, the levels of p62 were significantly decreased. A positive correlation was observed between LC3-II levels and systolic blood pressure, urinary protein concentration, and total urinary protein. Meanwhile, p62 levels were negatively correlated with systolic blood pressure. These results indicate that autophagy may participate in the pathological mechanism of pre-eclampsia (Pan et al., 2018). In the placenta of severe pre-eclampsia patients, the level of let-7i was decreased, and that of LC3-II was increased. *In vitro*, let-7i mimics can inhibit the autophagic activity of HTR-8 and JEG-3 cells (Xu et al., 2017). In the present study, increased LC3-II protein levels and LC3-II-specific immunofluorescence and decreased p62 protein levels in H/R-treated HUVECs. These findings suggest increased autophagy in HUVECs after induction of oxidative stress.

The SIRT protein family is a NAD^+^-dependent deacetylase and comprises seven members, namely, SIRT1–SIRT7. Their role in cell protection has gained increasing attention, and current research focuses on SIRT1 and SIRT3. SIRT1 is mostly distributed in the nucleus and affects oxidative stress responses, cellular metabolic process, autophagy, and apoptosis through deacetylation. As a mitochondrial protein, SIRT3 contributes to the regulation of the activity of proteases in mitochondria, which helps to maintain its stable state, and is related to selective autophagy. The upregulation of SIRT3 levels can inhibit mitochondrial fission through Drp1, reduce PINK1-mediated mitophagy, and avoid cellular dysfunction caused by massive mitochondrial degradation (Hu et al., 2022). Another research revealed that transient oxidative stress can activate SIRT3, inhibit cardiomyocyte apoptosis, and reduce myocardial injury by reducing ROS levels. However, when oxidative stress persisted, SIRT3 levels were downregulated, and a series of damages, such as ROS accumulation, decreased mitophagy activity, and increased apoptosis, occurred in cardiomyocytes (Song et al., 2021). The present work showed the involvement of SIRT3 in mitotic phagocytosis and apoptosis, and intracellular ROS level is a key factor for SIRT3 to perform such as regulatory function. SIRT3 levels were decreased in the placental tissues of patients with PIH and decreased in H/R-treated HUVECs. When pcDNA-SIRT3 was transfected to increase SIRT3 levels, LC3-II protein levels and LC3-II specific immunofluorescence were decreased, and p62 protein level was increased. Rapamycin, an autophagy activator, can counteract the inhibitory effect of SIRT3 on autophagy, which suggest that SIRT3 plays a protective role in autophagy triggered by oxidative stress.

Ferroptosis leads to cell death and is characterized by the accumulation of ferrous ions (Fe^2+^) and membrane damages caused by lipid peroxidation. The markers of ferroptosis include intracellular Fe^2+^ and lipid peroxide accumulation, increased ROS level, and GSH consumption. Mitochondrial damage and subsequent dysregulation of iron metabolism are important factors that promote the progression of diseases (Li et al., 2021). A strong causal link exists between them, that is, autophagy controls ferroptosis. The association between SIRT3 and dysregulation of iron metabolism has been reported. N-Acetylcysteine can maintain the redox balance of mitochondria by activating the SIRT3-superoxide dismutase (SOD) 2/GPX4 signaling pathway and then reduce damages caused by cellular ferroptosis in diabetic nephropathy (Li Q. et al., 2022). ANXA1sp can inhibit ferroptosis-induced cardiomyocyte death through the SIRT3-mediated deacetylation of p53, which protects it from sepsis-induced myocardial injury (Qin et al., 2023). In addition, the deubiquitinase USP11 regulates oxidative stress-induced ferroptosis by deubiquitinating and stabilizing SIRT3, which improves disc degeneration (Zhu J. et al., 2023).

PIH refers to a unique multisystem damage disease during pregnancy and is mainly characterized by systemic arteriolar spasm and inadequate perfusion of utero placenta, manifested by hypertension, urinary protein, etc. (Ristovska et al., 2023). A significant correlation exists between the occurrence of PIH and the ischemia and hypoxia of the uterus and placenta, vascular endothelial cell dysfunction, immune rejection, and coagulation dysfunction (Golovchenko et al., 2022). The most recognized etiologies of PIH include uterine placental ischemia and hypoxia. When one or several toxic factors produced by placenta enter the blood circulation, they will directly or indirectly cause vascular endothelial cell damage and trigger free radical production, which eventually leads to extensive circulatory dysfunction.

Lipid peroxidation and endothelial cell damage are common conditions in multiple pathological pregnancies. Patients with PIH are in a state of oxidative stress, with increases in oxygen free radicals and their peroxidation products and decreases in antioxidant components. A large number of free radicals can react with lipids in the cell membrane to produce lipid peroxidation products, especially oxidized low-density lipoproteins; these proteins can contract vascular endothelial cells and damage the morphological structure, especially the biofilm system, which results in endothelial cell dysfunction (Phoswa and Khaliq, 2021). The level of the lipid peroxidation metabolite MDA increased in maternal blood and umbilical cord blood of patients with PIH, which can be related to disease severity; the level of SOD, an important free-radical scavenger in the body, decreased significantly, which suggests a comprehensive decrease in the antioxidant protection function of patients with PIH (Yu and Zhou, 2022). In the present study, GSH content and GSH-Px activity were decreased, and the total iron and MDA contents were increased in the placental tissue of patients with PIH. This finding suggests that the progression of PIH is accompanied by ferroptosis. In H/R-treated HUVECs, the levels of free iron, MDA, and ROS and the colocalization of NCOA4 and LC3-II were increased, whereas the level of GSH was decreased. Under the action of pcDNA-SIRT3, H/R-induced ferroptosis was inhibited. Further studies revealed that H/R-induced ferroptosis was closely related to autophagy. Under the action of autophagy activator rapamycin, the inhibitory effect of SIRT3 on ferroptosis was eliminated. Our results indicate that SIRT3 alleviated H/R-induced injury by inhibiting autophagy-dependent ferroptosis.

The PI3K/Akt/mTOR signaling pathway is one of the main mechanisms that regulate autophagy and ferroptosis and may serve as a bridge between autophagy and ferroptosis. PI3K contributes to the formation of cell membrane, and its downstream target Akt transfers to the cell membrane after being activated by PI3K to phosphorylate its downstream target mTOR, which then mediates autophagy (Dai et al., 2023). Melatonin can inhibit ferroptosis in rat bone-marrow mesenchymal stem cells by activating the PI3K/Akt/mTOR signaling pathway and help alleviate the symptoms of osteoporosis induced by steroids (Li M. et al., 2022). In the progression of acute lung injury, tanshinone IIA can improve I/R-induced lung inflammation, ferroptosis, and apoptosis, which is mediated by the activation of the PI3K/Akt/mTOR pathway. When LY294002 was used to block PI3K signaling, the inhibitory effect of tanshinone IIA on iron metabolism dysregulation caused by I/R was counteracted (Zhang et al., 2023). In addition, the level of oxidative stress in the placental tissue of pre-eclampsia mice was decreased because of the activation of PI3K/Akt/mTOR pathway by mangiferin (Huang et al., 2020). Another research revealed that allethrin may induce excessive oxidative stress in the ovarian tissue of pregnant rats by inactivating the PI3K/Akt/mTOR signaling pathway (Jalouli et al., 2022). Although SIRT3 primarily functions within the mitochondria, its effects extend beyond this organelle. Through various indirect mechanisms, SIRT3 can influence key signaling pathways, including the PI3K/AKT/mTOR pathway, which regulate processes such as cell growth, proliferation, and autophagy. SIRT3 deacetylates and activates mitochondrial antioxidant enzymes, such as SOD2, reducing ROS production. Changes in ROS levels can act as signals to affect gene expression in the nucleus, for example, by modulating redox-sensitive transcription factors, such as nuclear factor-κB or activator protein 1, which indirectly influences the PI3K/AKT/mTOR signaling pathway (Mehranfard et al., 2023). In addition, SIRT3 can directly or indirectly regulate the activity of Forkhead box O (FOXO) family proteins (such as FOXO3a). FOXO proteins are involved in the regulation of autophagy-related gene expression and can promote autophagy by inhibiting mTORC1 (Wei et al., 2025). In summary, although SIRT3 primarily acts within the mitochondria, it exerts profound effects on the PI3K/AKT/mTOR signaling pathway through indirect mechanisms, such as modulation of ROS levels and transcription factors. Hyperhomocysteinemia (HHcy), a well-documented risk factor for cardiovascular diseases, such as hypertension, dysregulated autophagy in HUVECs. Elevated homocysteine levels can induce endoplasmic reticulum stress, mitochondrial dysfunction, and oxidative stress, all of which modulate the autophagic process. HHcy suppresses protective autophagy in endothelial cells, which contributes to endothelial dysfunction and vascular injury (Zhang et al., 2018; Yu et al., 2025). Intriguingly, SIRT3, a key mitochondrial deacetylase, has been implicated in counteracting oxidative and metabolic stress under HHcy conditions. Given that SIRT3 activity is often diminished in metabolic and cardiovascular stress states, HHcy may impair SIRT3 function, which leads to aberrant activation of the PI3K/AKT/mTOR pathway and suppression of autophagy. Thus, the SIRT3-mediated regulatory mechanism described in our study may represent a downstream effector pathway through which HHcy exerts its detrimental effects on endothelial homeostasis. Future studies investigating SIRT3 expression and activity in HHcy models will be essential to validate this hypothesis and further elucidate the molecular crosstalk between homocysteine metabolism and autophagy regulation.

In this study, the PI3K/Akt/mTOR pathway was blocked in H/R-treated HUVECs. During SIRT3 overexpression, the PI3K/Akt/mTOR signaling pathway was reactivated, which attenuated autophagy and iron metabolism dysregulation. However, under the action of PI3K inhibitor LY294002, this pathway was inhibited, and autophagy and ferroptosis were increased. One limitation of this study is the use of a single cell line for the experiments, which may limit the generalizability of our findings. Although this cell line has been widely used in related research, we plan to employ multiple cell models in future studies to further validate our conclusions. Moreover, although our findings demonstrate a clear functional link between SIRT3 and the regulation of the PI3K/AKT/mTOR signaling pathway and autophagy, the precise molecular mechanisms—particularly the potential epigenetic regulation through SIRT3-mediated histone deacetylation—remain to be fully elucidated. Although these epigenetic analyses were beyond the scope of the current study, we recognize their importance. We are currently designing follow-up experiments, including ChIP-qPCR and ChIP-seq, to explore the nuclear functions of SIRT3 and its potential association with specific genomic loci. These future research will help determine whether SIRT3 exerts direct transcriptional regulation over the pathways examined in this work, which will deepen our comprehension of its broader regulatory roles. In conclusion, our *in vivo* and *in vitro* studies confirmed that SIRT3 inhibited hypoxia-induced autophagy-dependent ferroptosis of HUVECs and alleviated PIH in mice by activating the PI3K/Akt/mTOR axis. Our results may provide new ideas for the treatment of PIH.

## Data Availability

The raw data supporting the conclusions of this article will be made available by the authors, without undue reservation.

## Glossary

PIH: Pregnancy-induced hypertension
SIRT: Sirtuins
HUVECs: Human umbilical vein endothelial cells
FBS: Fetal bovine serum
ROS: Reactive oxygen species
MDA: Malondialdehyde
GSH: Glutathione
GPX4: Glutathione peroxidase 4
MAP: Mean arterial pressure
H/R: Hypoxia/reoxygenation
SIRT3: Sirtuin 3
PI3K: Phosphoinositide 3-kinase
Akt: Protein kinase B
mTOR: Mechanistic target of rapamycin
SD: Sprague–Dawley
GD: Gestational day
NCOA4: Nuclear receptor coenzyme activator 4
PINK1: PTEN-induced putative kinase 1
DRP1: Dynamin-related protein 1
LC3: Light chain 3
